# Coupling between statistical learning and interference suppression

**DOI:** 10.1007/s00426-026-02375-6

**Published:** 2026-09-04

**Authors:** Kata Horváth, Dezso Nemeth, Karolina Janacsek, Andrea Kóbor

**Affiliations:** 1 Institute of Psychology, ELTE Eötvös Loránd University, Budapest, Hungary; 2 BML-NAP Research Group, Institute of Psychology, ELTE Eötvös Loránd University & Institute of Cognitive Neuroscience and Psychology, HUN-REN Research Centre for Natural Sciences, Budapest, Hungary; 3 INSERM, Université Claude Bernard Lyon 1, CNRS, Centre de Recherche en Neurosciences de Lyon CRNL U1028 UMR5292, Bron, France; 4 Gran Canaria Cognitive Research Center, Atlántico Medio University, Las Palmas de Gran Canaria, Spain; 5 Centre of Thinking and Learning, Institute for Lifecourse Development, School of Human Sciences, Faculty of Education, Health and Human Sciences, University of Greenwich, London, UK; 6 Brain, Memory and Language Research Group, Institute of Cognitive Neuroscience and Psychology, HUN-REN Research Centre for Natural Sciences, Magyar tudósok körútja 2, Budapest, H–1117 Hungary; 7 Brain Imaging Centre, HUN-REN Research Centre for Natural Sciences, Budapest, Hungary

## Abstract

In an ever-changing environment, synchronizing automatic and controlled behaviors is essential for successful adaptation. Yet, the relationship between these behaviors is often debated, partly because of the diverse approaches used to investigate them. The present study examined how statistical learning of environmental regularities, a process that supports automatic behaviors, and interference suppression, which contributes to controlled responding in the presence of distraction, interact when both operate concurrently. Participants completed a visual four-choice reaction time paradigm that combined a statistical learning task with the Eriksen flanker task. In this novel paradigm, a central target stimulus, surrounded by flanker stimuli, was either predictable or unpredictable based on the statistical regularities induced by the sequence of target stimuli. Statistical learning was indexed by the performance difference between predictable and unpredictable trials, whereas interference suppression was reflected by the flanker congruency effect (i.e., the performance difference between congruent and incongruent trials). Reaction times revealed a significant interaction: the flanker congruency effect was reduced on predictable trials, and the behavioral expression of statistical knowledge was larger in the incongruent than in the congruent condition. Together, these findings provide a proof-of-concept demonstration of a behavioral interaction between statistical learning and interference suppression within the present paradigm. Although this experimental design does not establish the causal direction of the interaction, the observed pattern indicates that learned predictability is associated with a reduced cost of conflicting distractors.

**Supplementary Information:**

The online version contains supplementary material available at https://doi.org/10.1007/s00426-026-02375-6.

## Introduction

Using automatic and controlled behaviors in tandem is essential for managing daily situations effectively. Luckily, the sensory, factual, and social information that surrounds us is structured into regularities that can be automatically learned (Conway, 2020; Frost et al., 2019). When relevant, we might use this knowledge to reduce the need for more effortful, controlled operations (Bugg, 2014; Decker et al., 2023; Egner, 2014; Melara & Algom, 2003). For instance, experienced readers can navigate a bookstore efficiently by exploiting typical shelving regularities. As another example, an experienced soccer player can execute a precise and rapid pass, even when confronted with opposing players advancing towards them. This can be derived from the regularity-based knowledge about the speed of the other players and the appropriate timing for initiating a pass, which has been repeatedly practiced. In the latter example, automatic behaviors may contribute to resolving conflicting options more efficiently, such as the decision to pass or hold the ball. Despite the prevalence of research on learning different types of environmental regularities, the question of how this learning mechanism contributes to a range of different behaviors remains unresolved (Bogaerts et al., 2020; Sherman et al., 2020). The present study employs a novel experimental design to contribute to this line of research by creating a situation in which automatic and controlled behaviors are engaged simultaneously.

Patterns and regularities hidden in the sensory input can be extracted in an unsupervised manner (Aslin, 2017; Conway, 2020; Frost et al., 2019, 2026). This process, known as statistical learning, contributes to the emergence of automatic behaviors (Ashby et al., 2010; Ullman, 2004). If needed, these automatic behaviors can be adjusted through different top-down control-based processes (Egner, 2014), commonly denoted as executive functions (e.g., Friedman & Robbins, 2022; Miyake et al., 2000). Efforts to unravel the relationship between statistical learning and executive functions have led to diverse theoretical approaches and experimental paradigms (e.g., Park et al., 2020, 2022; Pedraza et al., 2025; Smalle et al., 2022). Paradigms that combine the key features of statistical learning and executive functions simultaneously can uncover real-time interactions between these processes within a single experimental design. Studies following this approach are summarized next.

One line of research suggests that statistical learning and different executive functions can facilitate one another under certain conditions. In particular, it has been shown in various visual search tasks that statistical regularities embedded in the visual environment automatically drive attentional selection through target facilitation and distractor suppression (Duncan et al., 2023; Li & Theeuwes, 2020; Luck et al., 2021; Theeuwes et al., 2022). Additionally, the use of statistical regularities improves target detection in the absence of valid top-down guidance (i.e., pre-cueing, Dolci et al., 2023) and enhances post-perceptual decision efficiency regarding target presence (Wang et al., 2023). Statistical learning can also influence the selection of sensory input for conscious perception (Xu et al., 2024). Finally, in addition to exploiting the distributional probability of target and distractor features, statistical regularities across adjacent trial pairs also seem to support visual search (Li & Theeuwes, 2020). Nevertheless, beyond the interaction, independent operation of statistical learning and executive functions has also been observed in attentional (Gao & Theeuwes, 2020) and response (Jiménez et al., 2020a) selection.

Another line of research operationalizes statistical learning as extracting patterns or regularities spanning multiple trials, such as sequences (cf. Nissen & Bullemer, 1987). These sequences determine target presentation in tasks measuring executive functions. With these experimental designs, research has produced inconclusive evidence regarding whether executive control demands attenuate or enhance statistical learning. On the one hand, studies have shown that higher executive control demands impair statistical learning or the expression of statistical knowledge in modified search, Simon, and Go/NoGo tasks (Vaquero et al., 2020), as well as in oddball (Prutean et al., 2022) and complex visual search (Toh et al., 2021) paradigms. On the other hand, improved statistical learning or the improved expression of statistical knowledge has been found under high conflict induced by Stroop interference (Deroost et al., 2012; Takacs & Beste, 2023), by incompatible (Deroost & Soetens, 2006) or changing stimulus-response mappings (Deroost & Coomans, 2018), and by high perceptual load during visual search (Coomans et al., 2011). Altogether, considering previous research using different types of statistical regularities and executive functions, it is unclear whether and how statistical learning and executive functions interact when these processes are engaged simultaneously to facilitate adaptation.

In the present study, we focus on interference suppression, a component of inhibitory control. Inhibitory control is widely regarded as one of the core executive functions and a foundational process supporting higher-order cognition (Bari & Robbins, 2013; Friedman & Robbins, 2022). It encompasses multiple processes, including interference suppression and response inhibition (Bryce et al., 2011; Bunge et al., 2002). Interference suppression refers to the ability to select task-relevant information while resisting distraction from competing stimuli and is commonly assessed using conflict paradigms such as the Eriksen flanker and the Stroop tasks (Eriksen & Eriksen, 1974; Stroop, 1935). By contrast, response inhibition is typically measured with Go/NoGo and Stop-Signal paradigms (Logan et al., 1984; Verbruggen & Logan, 2008). In the Eriksen flanker task, participants must respond to target stimuli that are flanked by either congruent or incongruent distractors. Interference suppression is indexed by the flanker congruency effect, that is, poorer performance on incongruent than on congruent trials, which reflects the efficiency with which distracting information is resolved (Bryce et al., 2011; Brydges et al., 2012; Friedman & Miyake, 2004).

**Fig. 1:**
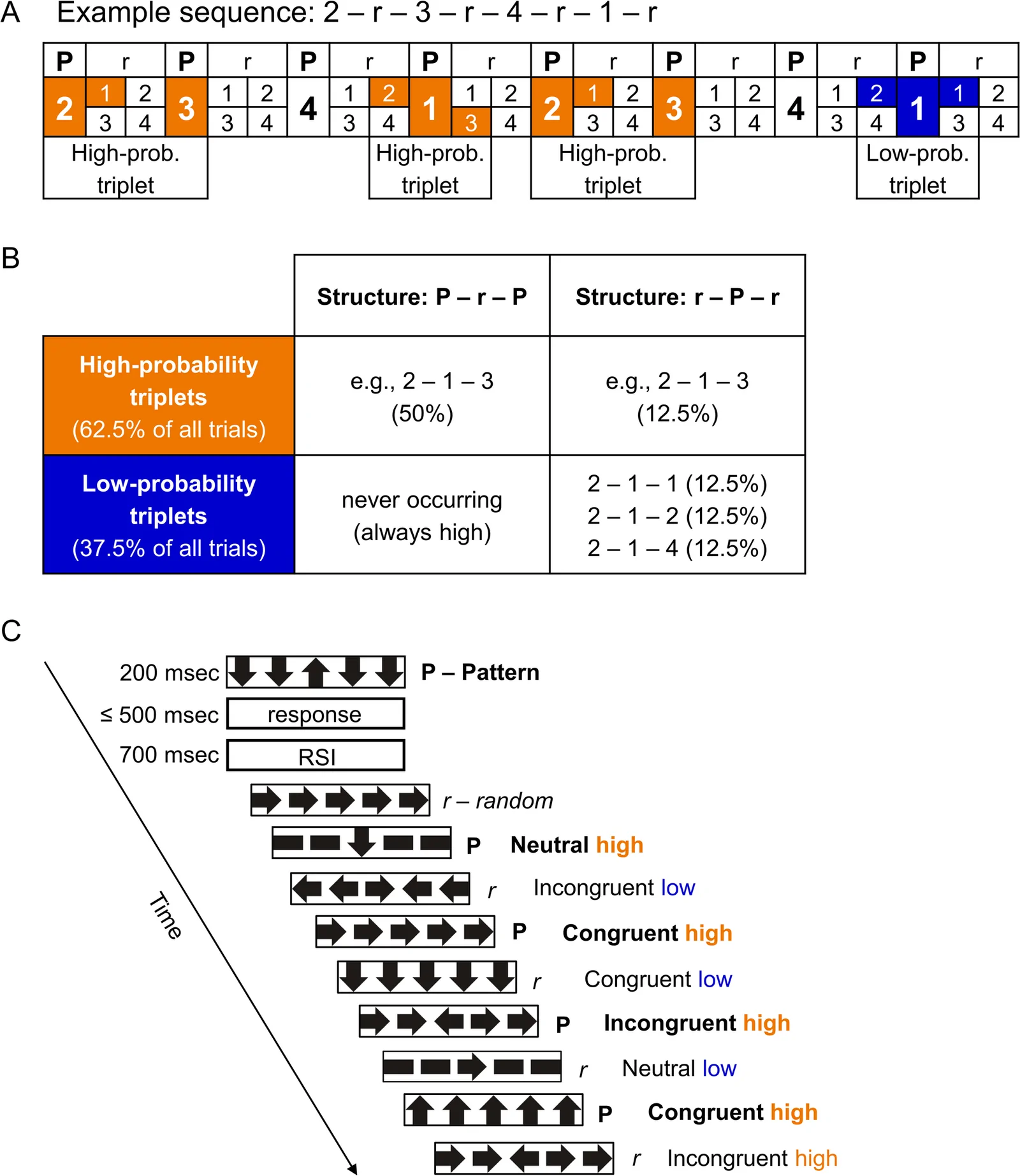
Design of the task.** (A)** An example of the sequence where predefined pattern (P) trials alternate with randomly selected (r) ones. Numbers represent the spatial direction of the target stimulus (1 = left, 2 = up, 3 = down, 4 = right). Pattern trials are represented with a single number corresponding to the example sequence. Random trials are represented by all four numbers, any of which can be selected randomly. **(B)** Due to the alternating nature of the sequence, some runs of three consecutive trials (triplets) occur with a higher probability and are thus predictable (high-probability triplets, 62.5% of all trials, marked by orange). Notably, high-probability triplets could also occur in the case of triplets with a random – pattern – random structure. Meanwhile, the occurrence of other random – pattern – random triplets is less probable in the task; therefore, these remain unpredictable (low-probability triplets, 37.5% of all trials, marked by blue). Blue and orange coloring indicates some examples of low- and high-probability triplets based on the example sequence. Probability type was defined in a moving window manner meaning that the third trial of every triplet was also the second trial of the next triplet and the first trial of the subsequent one. **(C)** Stimuli (central target and flankers together) were presented for a maximum of 200 msec, then, participants had a maximum of 500 msec to provide a response. Following a blank screen presented for 700 msec (response-to-stimulus interval, RSI), the next stimuli appeared on the screen. Trial-level flanker congruency was either congruent (37.5% of all trials), incongruent (37.5%), or neutral (25%), and was defined based on but not predicted by the alternating sequence. Each trial is categorized as the combination of trial-level probability and flanker congruency type. Note that there was only one possible incongruent flanker type for each target type, e.g., a left-arrow target was always paired with right-arrow flankers but not with up- or down-arrow flankers

Here, we combined an arrow version of the Eriksen flanker task (Stoffels & van der Molen, 1988) with a statistical learning task (Howard & Howard, 1997; Kóbor et al., 2019) into a visuomotor reaction time (RT) paradigm. Participants were asked to respond to the target (central) stimuli on the screen, which, unbeknownst to them, followed an alternating sequence (Fig. 1A). Due to the statistical regularities induced by the alternating sequence, each target stimulus was either predictable or unpredictable. This enabled us to assess statistical learning as the difference between responses to predictable vs. unpredictable trials (high- vs. low-probability triplets on Fig. 1B). At the same time, flanker stimuli also appeared on the screen as distractors surrounding the target (Fig. 1C). They were either congruent, incongruent, or neutral. The overall distribution of the flanker congruency types was equal across predictable and unpredictable trial types (see Methods). Crucially, suppressing distractor interference was necessary to ensure accurate responding to the targets. Altogether, this novel experimental design enabled us to test how statistical learning and interference suppression unfold within the same task, and whether these processes influence each other.

Beyond the interaction, it is also plausible that we would observe the independent operation of statistical learning and interference suppression in this experimental design because of the following reasons. First, to suppress interference from the surrounding peripheral distractors might be less demanding than resisting Stroop interference (Deroost & Coomans, 2018; Takacs & Beste, 2023), handling random trial-wise changes of stimulus-response mappings (Deroost & Coomans, 2018), or performing conjunction search (Toh et al., 2021). Hence, the contribution of statistical learning might not be needed to respond correctly to the target. Second, the statistical regularities span multiple trials in our paradigm, and the derived nonadjacent transitional probabilities are relatively complex (Conway, 2020). These differ from the statistical regularities used in most earlier studies (e.g., Deroost et al., 2012; Dolci et al., 2023; Duncan et al., 2023; Vaquero et al., 2020). Nevertheless, a wide range of studies demonstrated that, once acquired, this type of complex statistical knowledge is long lasting and resistant to interference originating from various experimental manipulations (e.g., Horváth et al., 2022; Kiss et al., 2022; Kóbor et al., 2017, 2020; Romano et al., 2010; Vékony et al., 2020b). Therefore, statistical learning could still emerge and remain unaffected by interference suppression.

The present study was exploratory in that we did not formulate a directional hypothesis regarding the relationship between statistical learning and interference suppression. Although the present paradigm has not been tested previously, prior work has demonstrated robust statistical learning under a variety of task demands, as reviewed above. Based on these previous findings, our primary expectation was that statistical learning would remain observable even when underlying regularities were embedded within the Eriksen flanker task. Our first aim was therefore to conduct a proof-of-concept test of this novel paradigm. Our second aim was to characterize the relationship between statistical learning and interference suppression when both processes are engaged within the same task.

If the two processes operated independently, we expected a similar statistical learning effect (i.e., faster RTs on predictable than on unpredictable trials) across all flanker congruency conditions, and a flanker congruency effect (i.e., slower RTs on incongruent than on congruent/neutral trials) of similar magnitude for predictable and unpredictable trials, resulting in no Triplet × Flanker interaction. If the two processes interacted, the resulting pattern could be consistent with either synergistic or competitive coupling. Given the mixed findings in the literature and the novelty of the present paradigm, we did not develop directional predictions regarding the nature of this interaction. In summary, the present study used a newly developed visuomotor reaction time paradigm to examine how statistical learning and interference suppression jointly shape adaptive performance.

## Methods

### Participants

The sample consisted of thirty-six participants (26 females and 10 males; *M*_Age_ = 22.4 years, *SD*_Age_ = 2.91 years, *M*_Education_ = 14.9 years, *SD*_Education_ = 1.89 years) who were recruited from university courses and via social media ads of our laboratory. To determine the required sample size for discovering a statistically significant interaction between statistical learning and interference suppression, we considered three different criteria. First, we conducted a *power analysis* based on two studies using a sequential Stroop task and thus matching the current design the closest. All analyses were carried out in G*Power 3.1 (Faul et al., 2007), using alpha = 0.05 and power = 0.90. Takacs and Beste (2023) observed a larger statistical learning effect in the incongruent condition of a sequential Stroop task compared with the neutral color-naming condition. Because they reported descriptive values as means and standard errors, we first converted the reported standard errors to standard deviations using the sample size of their Experiment 1. The effect size for the contrast between the incongruent and neutral color-naming conditions was then estimated assuming a correlation of *r* = .50 between repeated measures. This yielded Cohen’s *dz* = 0.97, indicating that a total sample of 14 participants would be sufficient to detect an effect of this magnitude. Deroost et al. (2012) found a similar pattern in their Experiment 3. Based on the reported condition means and standard deviations for the contrast between incongruent and congruent trials, and assuming a correlation of *r* = .50 between repeated measures, the estimated effect size was Cohen’s *dz* = 0.22. Detecting an effect of this magnitude with the above parameters would require an extremely large sample size of 220 participants. Therefore, based solely on the a priori sample size estimation from previous studies, we could not conclusively determine the sample size required to answer our main question.

Second, we considered *best practices* based on previous studies. In particular, we considered the study of Takacs and Beste (2023), in which 31 participants were analyzed in Experiment 1, and the study of Deroost et al. (2012), in which experimental groups comprised 13 to 36 participants, with a total of 45 participants in the aforementioned Experiment 3. In other studies that manipulated statistical-sequence learning and executive functions in a similar manner to the present one, the sample size varied from 13 to 36 per experiment or experimental group if applicable (Coomans et al., 2011; Deroost & Coomans, 2018; Deroost & Soetens, 2006; Jiménez et al., 2020a, b; Prutean et al., 2022). Furthermore, we considered recent studies of the Alternating Serial Reaction Time (ASRT) task (see Task and Procedure) where the original task was modified or combined with another paradigm (as in the present experiment). The sample sizes ranged from 23 to 33 subjects per experiment/group (Horváth et al., 2020, 2022; Kóbor et al., 2020; Vékony et al., 2020a, b). Therefore, we aimed for a sample size of at least 30 participants.

Finally, we took into account a *stopping rule* based on the flanker-ASRT sequence files used in the experiment (for more details, see Task and procedure and Supplementary Methods). Altogether, 12 unique sequence files were created which we aimed to repeat an equal number of times. Therefore, in line with all three considerations, we opted for a sample size of 36 participants.

All participants were healthy young adults, had normal or corrected-to-normal vision, and none of them reported a history of any psychiatric and/or neurological condition and substance use. Prior to their inclusion in the study, participants provided written informed consent to the procedure as approved by the United Ethical Review Committee for Research in Psychology (EPKEB) in Hungary. From the initially recruited sample, three participants were excluded for not meeting the above health criteria and another two due to technical errors; all were replaced to achieve a sample size of 36 participants. The study was conducted in accordance with the Declaration of Helsinki, and participants received course credits or supermarket vouchers (equivalent to a payment of ca. 5.5 euros) for taking part in the experiment.

### Task and procedure

The experiment consisted of one session. Participants performed a four-choice visuomotor RT task containing combined features of the Eriksen flanker task (Eriksen & Eriksen, 1974) and the ASRT task (Howard & Howard, 1997; Janacsek et al., 2012; Kóbor et al., 2019). In this combined task, a centrally presented arrow as the target stimulus appeared together with four flanker stimuli—two on the left and two on the right (Fig. 1). The flanker stimuli could be of three congruency types: pointing in the same direction as the target stimulus (congruent), pointing to the opposite direction as the target stimulus (incongruent), or having no spatial property (rectangular shapes, neutral). Four buttons of a response pad (Cedrus RB-540, Cedrus Corporation, San Pedro, CA) corresponded to the four possible spatial directions of the target stimulus (i.e., where the arrow pointed to). Participants were asked to press the button corresponding to the target stimulus (left = left thumb, up = left index finger, right = right index finger, down = right thumb) appearing on the screen as fast and as accurately as they could. Participants were instructed to focus only on the middle (target) stimulus and ignore the flanker stimuli.

Unbeknownst to the participants, the presentation of target stimuli followed an alternating sequence. In this sequence, predetermined pattern (P) trials alternated with randomly chosen ones (e.g., 2 – r – 3 – r – 4 – r – 1 – r, where numbers denote the four predetermined directions [1 = left, 2 = up, 3 = down, 4 = right], and *r*s denote the randomly chosen directions out of the possible four). Due to this alternation, some runs of three consecutive trials (triplets) occurred with a higher probability than others and thus were predictable. For all triplets, the third element (*n*) could be predicted by the first element (*n* – 2) of that triplet with either high or low probability, while the middle element (*n* – 1) did not have a predictive value. In the example above, 2 – x – 3, 3 – x – 4, 4 – x – 1, and 1 – x – 2 (where x denotes the middle element of a triplet) were more probable, as these were presented in every sequence repetition and also by chance. Meanwhile, for instance, 2 – x – 1, 3 – x – 2, 4 – x – 3, and 1 – x – 4 occurred with a lower probability as these triplets could only be formed by chance and thus were unpredictable. We refer to the former category as high-probability or predictable triplets (62.5% of all trials) and as low-probability or unpredictable triplets to the latter (37.5% of all trials). Triplets were identified using a moving window throughout the stimulus stream; thus, each trial was categorized as the last element of a high- or low-probability triplet; then, the same trial served as the middle and the first element for the categorization of the following triplets (Fig. 1).

The task was organized into blocks, each consisting of 85 trials. In the first five trials, randomly chosen arrows were presented and served as a warm-up; this was followed by ten repetitions of the eight-element alternating sequence. Each stimulus was presented as the combination of a central target stimulus and four flanker stimuli. The flanker congruency of the five warm-up trials was randomly selected, whereas the congruency of each trial of the repeating sequence was predetermined based on but not directly predicted by the nonadjacent probability structure of the alternating (ASRT) sequence. In more detail, flanker congruency was defined for the last element of every unique triplet (e.g., 2 – 1 – 3 or 1 – 3 – 1 following the example above; 64 altogether). For all occurrences of a given unique triplet, the last element had the same flanker congruency consistently. 37.5% of both high-probability and low-probability triplets were assigned into the congruent and incongruent flanker congruency types, while the remaining 25% were assigned into the neutral type. For further information on how we assigned flanker congruency types onto the probability structure of the task, see the Supplementary Methods.

Stimuli—the combined target-flanker stimulus array—were presented centrally on the screen for 200 msec. Then, a blank screen was presented until a response was provided but no longer than 500 msec. Following this, another blank screen was presented for 700 msec (response-to-stimulus interval, RSI); then, the next stimuli appeared. Participants could also respond during stimuli presentation; in this case, the stimuli disappeared from the screen after response onset, and only the RSI blank screen was presented. In the case of an incorrect response, 500 msec after incorrect response onset, a black “X” was presented for another 500 msec. In the case of no response within the given time window, an “!” was presented for 500 msec. RTs were calculated from stimuli onset.

The experiment consisted of 30 blocks altogether, following a two-block-long practice phase where all stimuli appeared in a random order (Kóbor et al., 2019). A short self-paced break was administered before each block started. The task was implemented using Presentation software (Version 21.1; Neurobehavioral Systems, Inc., Berkeley, CA). Stimuli were displayed on a 21-in. LCD screen at a viewing distance of 125 cm. After completion of the task, a short post-task questionnaire was assessed. Participants were asked to report anything special observed and/or any regularity discovered in the task. None of them reported any regularity possibly linked to the alternating (ASRT) sequence. However, because sequence awareness was assessed only with an open-ended post-task questionnaire, which may provide a relatively insensitive measurement of explicit knowledge (Lovibond & Shanks, 2002; Vadillo et al., 2016), the present data do not permit firm conclusions regarding the explicit or implicit status of the acquired knowledge.

### Statistical analysis

Trial types were defined by the combination of probability (high vs. low) and flanker congruency (congruent, incongruent, or neutral). Before analysis, the original trial set was filtered using the following criteria. First, the five warm-up trials at the beginning of each task block were excluded, as they were not part of the eight-element alternating sequence. Second, trials with RTs below 100 msec were removed to eliminate rapid, impulsive responses; this also involved excluding missing responses with an RT of 0 msec. Third, following the standard data analysis protocol of the ASRT task, two categories of low-probability triplets were excluded: trills (e.g., 1 – x – 1 or 3 – x – 3) and repetitions (e.g., 2 – 2 – 2 or 3 – 3 – 3; Nemeth et al., 2013a; Song et al., 2007), as these are influenced by pre-existing response tendencies (Howard et al., 2004). Fourth, high-probability triplets with random – pattern – random structure (Fig. 1) were also removed (Horváth et al., 2021; Kóbor et al., 2021). These “accidentally regular” random triplets are formally predictable yet occur infrequently at the level of unique triplets (Kóbor et al., 2019), and they have been shown to elicit response biases stemming from higher-order structure in the alternating sequence (Szegedi-Hallgató et al., 2019). Excluding them ensured that analyses contrasted genuinely predictable pattern triplets with genuinely unpredictable random triplets (i.e., pattern high-probability vs. random low-probability triplets) when quantifying statistical learning. Finally, incorrect responses were removed from the analysis of RTs. Because every trial belongs to a triplet, the terms “trial” and “triplet” are used interchangeably; thus, excluding a single triplet is equivalent to excluding a single trial.

A linear mixed-effects model was fitted to RTs using the *lmer* function from the *lme4* package (Bates et al., 2015) in R (R Core Team, 2020). The fixed effects included *Triplet* (high vs. low), *Flanker* (neutral, congruent, incongruent), and their two-way interaction. Both predictors were treated as categorical variables. Random intercepts were included for each participant × block combination. Model parameters were estimated using restricted maximum likelihood (REML). *p*-values for fixed effects were obtained using Satterthwaite’s degrees of freedom approximation implemented in the *lmerTest* package (Kuznetsova et al., 2017). Sum coding was applied, with *low* (Triplet) and *incongruent* (Flanker) set as reference levels. Pairwise comparisons were conducted using the *emmeans* package (Lenth, 2022) with the Tukey method for *p*-value adjustments. Figures were generated with the *ggplot2* package (Wickham, 2016). The schematic form of the model is shown below, with the dependent variable on the left of the “~” symbol, the predictors and random intercepts on the right: RTs ∼ Triplet × Flanker + (1 ∣ participant: block).

Error rate was generally below 7%, except for the incongruent trials (see Table S5 in the Supplementary Results), indicating an overall accurate performance and a near-ceiling effect, commonly observed in the ASRT task (e.g., Kóbor et al., 2019; Vékony et al., 2020a). Therefore, we focus on the analysis of RTs in the main text and present the analysis of error rates in the Supplementary Results. The conclusions of the error rate analysis are summarized below in the Results section.

In flanker tasks, sequential trial effects are often accounted for to isolate conflict-driven adaptation processes (Braem et al., 2019; Egner, 2007; Mayr et al., 2003; Nieuwenhuis et al., 2006; Schmidt, 2019). Because statistical regularities in our novel task span multiple trials, we aimed to ensure that other types of sequential effects did not influence the potential interaction between statistical learning and flanker congruency. To this end, we fitted an additional linear mixed-effects model to RTs controlling for repetition priming (feature repetition). Specifically, beyond triplet type and flanker congruency, each trial *n* was classified according to its feature overlap with trial *n* − 1: We coded whether the trial pair involved distractor repetition or distractor change (flanker stimuli pointing in the same or different direction across trials) and response repetition or response change (central stimulus requiring the same or different response across trials). This analysis was considered exploratory, and the details and full results are presented in the Supplementary Results. The conclusions of the repetition priming analysis are summarized below in the Results section.

## Results

### RTs

The linear mixed-effects model (see Table 1) revealed significant statistical learning (main effect of Triplet), with faster responses for predictable than unpredictable trials (β = − 3.02, *SE* = 0.25, *t* = − 11.92, *p* < .001). This statistical learning effect was approximately 6 msec in magnitude (high-probability triplets: *M* = 371 msec [*SE* = 0.97]; low-probability triplets: 377 msec [*SE* = 1.02]). A strong flanker congruency effect emerged as well (main effect of Flanker). Relative to incongruent trials, responses were significantly faster on both neutral (β = − 8.64, *SE* = 0.38, *t* = − 22.56, *p* < .001) and congruent trials (β = − 11.29, *SE* = 0.34, *t* = − 32.72, *p* < .001), corresponding to RT reductions of approximately 29 msec and 31 msec, respectively (neutral trials: *M* = 365 msec [*SE* = 1.05]; congruent trials: *M* = 363 msec [*SE* = 1.01]; incongruent trials: *M* = 394 msec [*SE* = 1.02]). Congruent trials were characterized by significantly faster RTs relative to neutral ones (*t* = 4.13, *p* < .001).

**Table 1:** Summary of the linear mixed-effects model testing the effects of predictability and flanker congruency on RTs

Terms	β	*SE*	*95% CI*	*t*	df	*p*
(Intercept)	373.920	0.963	372.030–375.810	388.164	1099.594	< .001
Triplet (High vs. Low)	-3.018	0.253	-3.515 – -2.522	-11.918	58716.526	< .001
Neutral vs. Incongruent	-8.644	0.383	-9.395 – -7.893	-22.564	58798.787	< .001
Congruent vs. Incongruent	-11.286	0.345	-11.962 – -10.609	-32.716	58774.269	< .001
Triplet × Neutral vs. Incongruent	0.025	0.382	-0.725–0.774	0.064	58772.547	.949
Triplet × Congruent vs. Incongruent	0.835	0.346	0.157–1.513	2.415	58807.928	.016
Random Effects						
σ^2^	3288.925					
τ_00 ID: block_	932.839					
ICC	0.221					
N _ID_	36					
N _block_	30					
Observations	59,753					
Marginal R^2^ / Conditional R^2^	0.048 / 0.258					

Note. Coding scheme: sum coding. Reference levels: low (Triplet) and incongruent (Flanker). β estimates correspond to the sum-coded model parameters; pairwise comparisons reported in the text are based on estimated marginal means. *SE*: standard error; *CI*: confidence interval

Statistical learning effects were significantly above zero in all flanker congruency conditions (all *p*s < .001; 5.99 msec, 4.37 msec, and 7.76 msec for the neutral, congruent, and incongruent trials, respectively), indicating robust statistical learning. Crucially, however, the Triplet × Flanker interaction was significant. This interaction can be described relative to either statistical learning or flanker congruency effects. First, the statistical learning effect was larger for the incongruent trials than for the congruent ones (β = 0.84, *SE* = 0.35, *t* = 2.42, *p* = .016; difference = 3.39 msec; *t* = − 2.91, *p* = .010). By contrast, the size of the statistical learning effect did not differ significantly between incongruent and neutral trials (β = 0.02, *SE* = 0.38, *t* = 0.06, *p* = .949; difference = 1.77 msec; *t* = − 1.37, *p* = .359) or between congruent and neutral trials (difference = 1.62 msec; *t* = 1.27, *p* = .413).

**Fig. 2:**
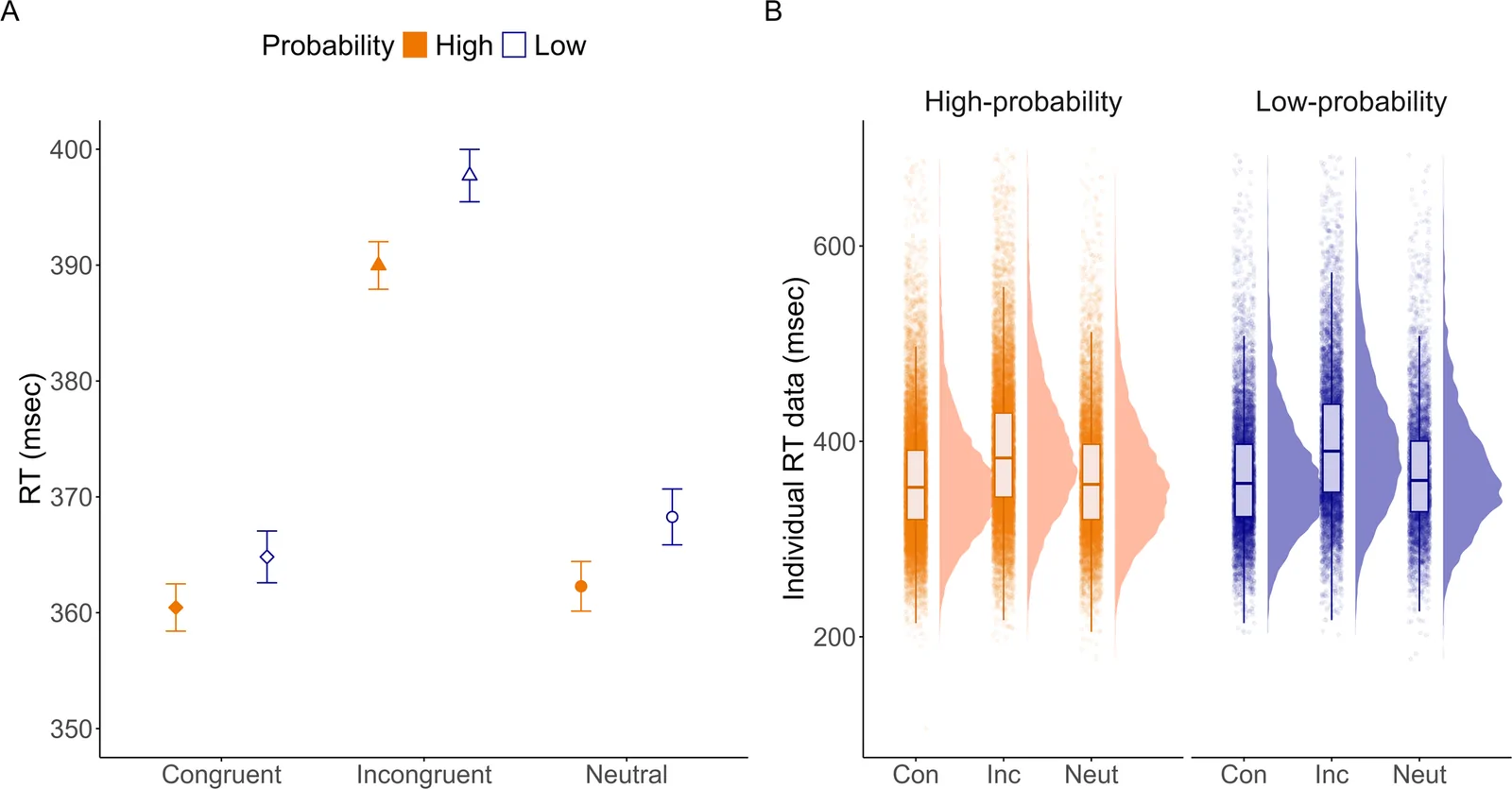
Main task performance across probability and flanker congruency conditions.** (A)** Mean RTs across probability conditions, with orange representing high-probability (predictable) trials and blue representing low-probability (unpredictable) trials, shown separately for each flanker congruency type on the x-axis (congruent, incongruent, neutral). RTs were faster for high-probability than for low-probability trials, reflecting statistical learning. RTs were slower on incongruent than on congruent or neutral trials, reflecting the flanker congruency effect. In addition, probability and flanker congruency interacted significantly. Error bars denote 95% confidence intervals. **(B)** Individual trial-level RTs (jittered, semi-transparent points), overlaid with half-eye density plots (colored areas) representing the distribution of RTs across probability and flanker congruency conditions (Con = congruent, Inc = incongruent, Neut = neutral). Central boxplots indicate the median (horizontal line) and interquartile range (box edges), with whiskers extending to 1.5 times the interquartile range

Second, the flanker congruency effect (incongruent > congruent) was smaller on predictable relative to unpredictable trials. In particular, the flanker congruency effect was 29.52 msec for predictable and 32.91 msec for unpredictable trials (difference = 3.39 msec; *t* = − 2.91, *p* = .010). Relative to neutral trials, incongruent flankers delayed responses by 27.69 msec on predictable trials (*t* = − 36.86, *p* < .001) and by 29.46 msec on unpredictable trials (*t* = − 27.91, *p* < .001). However, the 1.77-msec difference in interference between predictable and unpredictable trials was not significant (*t* = − 1.37, *p* = .359). Similarly, relative to neutral trials, congruent flankers speeded responses by 1.83 msec on predictable trials (*t* = 2.46, *p* = .037) and by 3.45 msec on unpredictable trials (*t* = 3.32, *p* = .003). The 1.62-msec difference in facilitation was also not significant (*t* = 1.27, *p* = .413). Thus, the smaller flanker congruency effect on predictable trials was not attributable to a significant difference in either interference or facilitation considered separately; rather, both components were numerically smaller on predictable trials, and their combined difference was reflected in the significant difference in the overall flanker congruency effect. These results are presented in Fig. 2.

### Accuracy (error rate)

Participants made fewer errors on predictable than on unpredictable trials (5.5% vs. 7.0%), demonstrating robust statistical learning (*p* < .001). A strong flanker congruency effect also emerged, with the lowest error rates on congruent trials (4.3%), higher on neutral trials (6.0%), and the highest on incongruent (9.7%) ones (all *p*s < .001). Unlike RTs, error rates showed no significant Triplet × Flanker interaction (both *p*s ≥ .328), indicating that statistical knowledge was similarly expressed across the flanker congruency conditions as well as that the flanker congruency effect did not differ between predictable and unpredictable trials. Importantly, statistical learning effects were significant within each flanker congruency condition (all *p*s < .001). (See Supplementary Results, Table S6, and Figure S1 for details.)

### Analysis of RTs controlling for repetition priming

Even after accounting for distractor and response repetition, participants showed significant statistical learning and flanker congruency effects (all *p*s < .001). Crucially, the Triplet × Flanker interaction remained significant (β = 1.48, *SE* = 0.47, *t* = 3.14, *p* = .002), and was numerically stronger than in the simpler model. Thus, short-term repetition priming alone is unlikely to account for the interaction. The combination of distractor and response repetition modulated the flanker congruency effects (both *p*s < .001). Furthermore, response repetition reduced the statistical learning effect (*p* = .012). Importantly, however, none of these influences undermined the Triplet × Flanker interaction. (See Supplementary Results, Tables S7–S9, and Figure S2 for details.)

## Discussion

We examined whether and how statistical learning and interference suppression interact during adaptive performance. Based on our results, the behavioral expression of statistical knowledge was larger in the incongruent condition, which was also reflected in a reduced flanker congruency effect on predictable relative to unpredictable trials. Altogether, this pattern shows a behavioral interaction between statistical learning and interference suppression within a novel paradigm.

Although the current paradigm permits characterization of the interaction between statistical learning and interference suppression, it does not allow us to draw conclusions about the direction of influence between the two processes. The observed pattern nevertheless allows for two possible interpretations. One possibility is that target predictability contributed to more efficient performance under interference, particularly in the incongruent condition (cf. Deroost et al., 2012). In this account, participants may have relied more strongly on predictable target information when resolving distractor conflict. Similar interpretations have been proposed in visual search paradigms, where statistical regularities facilitate performance in situations involving different top-down control-based processes (Duncan et al., 2023; Li & Theeuwes, 2020; Theeuwes et al., 2022; Xu et al., 2024). Notably, however, the decomposition of the flanker congruency effect indicated that neither interference from incongruent flankers nor facilitation from congruent flankers differed significantly between predictable and unpredictable trials when considered separately. Rather, both components were numerically smaller on predictable trials, and their combined difference was reflected in the overall interaction. Altogether, the present findings are consistent with the possibility that statistical regularities become increasingly behaviorally relevant under conditions involving distractor interference (cf. Bugg, 2014; Takacs & Beste, 2023).

An alternative interpretation is that increased conflict in the incongruent condition amplified the behavioral expression of statistical knowledge, consistent with several earlier studies reporting larger statistical learning effects under increased control demands (e.g., Coomans et al., 2011; Deroost & Soetens, 2006; Takacs & Beste, 2023). One possible account of this pattern is provided by the adaptation by binding theory (Verguts & Notebaert, 2009), according to which conflict-related arousal strengthens task-relevant representations through associative learning. In the present paradigm, predictive information derived from statistical regularities may have increased the activation of target representations, and this activation may have been further strengthened under incongruent conditions characterized by elevated conflict. Importantly, however, the present behavioral data do not allow us to determine whether the observed interaction primarily reflects the contribution of predictability to interference resolution, increased expression of statistical knowledge under conflict, or a combination of both. Future studies using structurally different combined paradigms may help disentangle these possibilities. For instance, in a between-subjects design, statistical learning and interference suppression could occur in separate phases at the beginning of the task; then, their influence on one another could be tested in a combined phase where both processes should work together (cf. Deroost et al., 2012).

Notably, the present findings differ from several previous studies in which increased executive control demands impaired statistical learning or reduced its behavioral expression (Bocanegra & Hommel, 2014; Prutean et al., 2022; Stock et al., 2016; Toh et al., 2021; Vaquero et al., 2020; Zink et al., 2018). These earlier findings may be interpreted within frameworks proposing competitive relationships between statistical learning and executive functions. One influential account is the competitive memory systems framework, which originally postulates a competitive-antagonistic relationship between the basal ganglia-dependent and the prefrontal/medial temporal lobe-dependent learning and memory processes (Albouy et al., 2013; Freedberg et al., 2020; Poldrack et al., 2001; Poldrack & Packard, 2003). This framework has been extended to proposing competition between statistical learning and executive functions/inhibitory control (e.g., Albouy et al., 2013; Ambrus et al., 2020; Borragán et al., 2016; Conway, 2020; Park et al., 2022; Pedraza et al., 2024; Takacs & Beste, 2023; Virag et al., 2015). In line with this, several studies reported enhanced statistical learning after executive functions had been experimentally attenuated using interventions such as noninvasive brain stimulation, hypnosis, or cognitive fatigue/depletion induced by dual working-memory tasks (Ambrus et al., 2020; Borragán et al., 2016; Nemeth et al., 2013b; Pesthy et al., 2025; Smalle et al., 2021, 2022).

The current results should be distinguished from these intervention-based findings in important respects. First, no executive functions were experimentally reduced or disrupted in the present paradigm. Instead, interference suppression constituted an integral component of successful task performance. Second, the observed interaction emerged while participants successfully resolved distractor interference (i.e., targets were responded to correctly), rather than following manipulations designed to attenuate executive functions. Consequently, the current data do not straightforwardly fit a strong antagonistic interpretation in which reduced executive functions directly facilitate statistical learning.

At the same time, the present findings do not necessarily rule out competition-based accounts entirely. One possibility is that the parallel engagement of interference suppression changed how strongly statistical knowledge influenced performance under distractor conflict, without implying reduced inhibitory control per se. More generally, the relationship between statistical learning and executive functions may depend on the specific inhibitory control subprocess involved, as well as on task demands and attentional state. In this respect, recent evidence combining the ASRT task with a Go/NoGo task may be relevant. One study found that weaker response inhibition was related to enhanced statistical learning, with this relationship being more pronounced during periods of mind wandering, when attention deviated from the task (Vékony et al., 2025a). This finding is consistent with other evidence suggesting that mind wandering may enhance statistical learning (cf. Simor et al., 2025; Vékony et al., 2025b). Another study using the same combined ASRT–Go/NoGo task similarly found an association between weaker response inhibition and stronger statistical learning, while also showing that the strength of this relationship varied as a function of ADHD-like traits (Horváth et al., 2026). Although these studies focused on response inhibition rather than interference suppression, they suggest that the relationship between statistical learning and inhibitory control may differ across paradigms, attentional states, and individuals.

Altogether, the present findings indicate that statistical learning and interference suppression can operate concurrently to support adaptive behavior within the same task context. By contrast, competition-related patterns reported in earlier studies may emerge particularly in paradigms involving experimentally reduced executive functions. Clarifying when different relationships arise will require future studies directly comparing different executive control demands, task structures, attentional states, and intervention-based manipulations.

The diversity of findings across the literature may partly reflect substantial methodological differences in how the relationship between statistical learning and executive functions has been investigated. Earlier studies can be broadly grouped into four categories. The first category includes behavioral studies that measured statistical learning and executive functions with separate tasks and examined correlations between their performance indices (e.g., Brezóczki et al., 2026; Éltető et al., 2022; Janacsek & Nemeth, 2013; Park et al., 2020; Pedraza et al., 2024; Pedraza et al., 2025; Virag et al., 2015). These studies reported positive, negative, and null associations, suggesting that the relationship between the two processes may vary across individuals and task contexts. Importantly, this line of research primarily addresses the relationship between trait-like individual differences in statistical learning and executive functions, unlike the following categories that focus more directly on state-like interactions during task performance (as exceptions, see Lum et al., 2023a; Virag et al., 2015).

The second category includes studies in which executive functions were experimentally depleted, disrupted, or suppressed using various interventional methods (Ambrus et al., 2020; Borragán et al., 2016; Nemeth et al., 2013b; Pesthy et al., 2025; Smalle et al., 2021, 2022). Many of these studies reported enhanced statistical learning following the reduction of executive functions, although exceptions have also been observed (Janacsek et al., 2015; Thompson et al., 2014). Relatedly, a third category of studies linked improved statistical learning to reduced engagement of brain networks associated with executive functions, without directly manipulating these networks (Lum et al., 2023a, b; Park et al., 2022; Simor et al., 2025; Tóth et al., 2017; Vékony et al., 2025b).

Finally, the fourth category includes combined paradigms, such as the present study, those reviewed in the Introduction, and the task developed by Vékony et al. (2025a) (see also Horváth et al., 2026), in which statistical learning and executive functions are recruited simultaneously. These paradigms offer a unique opportunity to examine real-time interactions between the two processes during ongoing performance. At the same time, as illustrated by the present findings, such designs may make it difficult to determine the causal direction of influence between statistical learning and executive functions.

One methodological limitation of the present paradigm concerns the fixed mapping between unique triplets and flanker congruency types. Although flanker congruency was not directly predictable from the nonadjacent transitional probability structure that determined triplet probability, the congruency of the third element was held constant for each unique triplet throughout the experiment. Consequently, participants could in principle learn additional triplet-specific congruency contingencies. Because individual high-probability triplets occurred more frequently than individual low-probability triplets, participants also had more opportunities to acquire these contingencies for high-probability triplets. Such differential learning could have facilitated preparation for the upcoming flanker congruency and may therefore have contributed to the observed Triplet × Flanker interaction. The present design does not allow this alternative account to be ruled out. Future studies should orthogonalize unique-triplet identity and flanker congruency, such that each unique triplet occurs across the different congruency conditions with approximately equal frequency, thereby separating statistical predictability from triplet-specific congruency learning.

A separate, broader methodological consideration concerns the correlated structure of the present paradigm, which could potentially explain the observed interaction. Our paradigm can be conceptualized as a variant of a correlated flanker task, in which target and flanker stimuli are processed simultaneously and may become integrated during performance (e.g., Decker et al., 2023; Landau et al., 2012). In the present design, conflict emerged between flankers and targets in the incongruent condition, while the targets themselves followed learnable statistical regularities across multiple trials. It is possible that these concurrent regularities promoted more integrated processing of the entire stimulus array.

More importantly, certain task features were not fully independent. Each target direction was paired with only one incongruent flanker configuration (see Fig. 1), and stimulus-response mappings were spatially compatible (e.g., the left-arrow stimulus corresponded to the left-orienting response key). Therefore, participants may have incidentally acquired contingent relationships among targets, flankers, and responses, which could have facilitated responding correctly. Future studies could address this possibility more directly by systematically manipulating probabilistic relations between flankers and targets (Carlson & Flowers, 1996; Decker et al., 2023; Landau et al., 2012; Miller, 1987).

Other types of correlations between task features (Braem et al., 2019; Nieuwenhuis et al., 2006) may also have contributed to the observed interaction. However, the interaction remained reliably detectable after accounting for feature repetition, i.e., stimulus-response priming or repetition priming. This suggests that the interaction is unlikely to be derived merely from short-term sequential regularities across trials, such as the repetition of distractors and/or responses. Of note, independence between statistical learning and executive functions was still observed in another study where perceptuomotor features of the previous trial predicted congruency features of the current trial, yielding correlated stimulus/response dimensions (Jiménez et al., 2020a). Overall, although the present analysis argues against a simple repetition-priming account of the interaction, it does not rule out contributions from the broader task contingencies described above, particularly the fixed mappings between unique triplets and flanker congruency.

## Conclusions

Our findings provide proof-of-concept evidence that statistical learning and interference suppression can be jointly examined within a single task and that their behavioral effects interact during performance. At the same time, the present design does not allow us to determine the direction of influence underlying this interaction and cannot rule out a contribution from additional learned task contingencies, particularly triplet-specific congruency learning. Future research should orthogonalize triplet identity and flanker congruency, while combined and temporally segregated paradigms as well as neurocognitive measures will be important for clarifying the directionality and mechanisms underlying the interaction.

## Supplementary Information

Below is the link to the electronic supplementary material.

Supplementary Material 1 (PDF 0.98 MB)

## Data Availability

The dataset supporting the conclusions of this article is available via the Open Science Framework (https://osf.io/nx73e/files) in the fASRT_behavioral_data folder. The experiment was not preregistered.

## Declarations

### Generative AI and AI-assisted technologies in the writing process

During the preparation and revision of this manuscript, the authors used the AI-based tool ChatGPT (OpenAI) for language editing and stylistic refinement of the English text. The tool was not used for data analysis, statistical testing, or the generation of scientific conclusions. All outputs were critically reviewed, revised, and verified by the authors, who take full responsibility for the content of the publication.

### Public Significance Statement

This study shows that picking up patterns from the environment is associated with more efficient responding when distracting information is present. In this task, people are less affected by conflicting distractors when upcoming events are predictable. These findings suggest that knowledge acquired automatically through experience can support performance in challenging situations and provide new insight into how humans adapt effectively to complex environments.
