# Supplementary material for: Coupling between statistical learning and interference suppression
Source: Psychol Res. 2026 Sep 4;90(5):160. doi: 10.1007/s00426-026-02375-6 (PMC13545042; doi:10.1007/s00426-026-02375-6)
Supplement: Supplementary file 1 [file 426_2026_2375_MOESM1_ESM.pdf]

Supplementary Information for "Coupling between statistical learning and interference suppression"

K. Horváth, D. Nemeth, K. Janacsek, A. Kóbor

Table of Contents

1. Supplementary Methods ... 2
1.1 Assignment of flanker congruency and selection of the flanker-ASRT sequences ... 2
Table S1. Main effects and interactions observed in the log-linear regression for congruent vs. incongruent high-probability trials. ... 5
Table S2. Main effects and interactions observed in the log-linear regression for congruent vs. incongruent low-probability trials. ... 6
Table S3. Main effects and interactions observed in the log-linear regression for neutral high-probability trials. ... 6
Table S4. Main effects and interactions observed in the log-linear regression for neutral low-probability trials. ... 6
2. Supplementary Results ... 7
Table S5. Estimated marginal means: Accuracy (error rate). ... 7
2.1 Analysis of accuracy (error rate) ... 7
Table S6. Summary of the logistic mixed-effects model testing the effects of predictability and flanker congruency on errors. ... 9
2.2 Analysis of RTs controlling for repetition priming ... 10
Table S7. Summary of the linear mixed-effects model testing the effects of predictability, flanker congruency, and repetition priming on RTs. ... 13
Table S8. Follow-up of the Flanker × Distractor Repetition × Response Repetition interaction: Pairwise comparisons of the flanker congruency effects (Incongruent vs. Congruent, Incongruent vs. Neutral) across the factor level combinations of repetition priming (Distractor Repetition × Response Repetition). ... 16
Table S9. Follow-up of the Flanker × Distractor Repetition × Response Repetition interaction: Pairwise comparisons of each flanker type (Neutral, Congruent, and Incongruent) across the factor level combinations of repetition priming (Distractor Repetition × Response Repetition). ... 17
Supplementary References ... 19

1. Supplementary Methods

1.1 Assignment of flanker congruency and selection of the flanker-ASRT sequences

Although the underlying alternating sequence and the nonadjacent triplet structure did not directly predict flanker congruency, flanker congruency was pre-assigned to all trials to ensure counterbalancing across probability types. To do so, unique triplets (e.g., 1 [left] – 3 [down] – 2 [up]) were considered instead of the predictive associations (e.g., 1 [left] – x [randomly selected out of the four possible] – 2 [up]) between the first and the last element of either a high- or low-probability triplet. In the ASRT task, while predictability (i.e., whether a triplet (trial) is high- or low-probability) is determined by the association of a triplet's first and last element, unique triplets can be identified if all three elements are considered. For each predictive association, there are altogether four unique triplets with the four possible spatial directions as the middle element.

The assignment process involved the following steps: 1) identifying unique triplets within the ASRT sequences, 2) separately assigning the flanker congruency types to high-probability and low-probability triplets, and 3) creating additional assignment variations by repeating step 2. The selection process entailed statistically inspecting the generated variations of the combined flanker congruency and probability structure and excluding suboptimal variations.

Assignment – As the first step of assigning the flanker congruency types onto the probability structure of the ASRT sequences, unique triplets were identified. According to the possible permutations of the four spatial directions (4! = 24) and the continuous presentation of the sequence (i.e., the starting element of the sequence does not affect the sequence order and the probability structure), altogether six unique (base) sequences can be identified (1 – r – 2 – r – 3 – r – 4 – r, 1 – r – 2 – r – 4 – r – 3 – r, 1 – r – 3 – r – 2 – r – 4 – r, 1 – r – 3 – r – 4 – r – 2 – r, 1 – r – 4 – r – 2 – r – 3 – r, and 1 – r – 4 – r – 3 – r – 2 – r). Within each sequence, altogether 64 unique triplets can be distinguished (4 [first position] × 4 [second position] × 4 [third position] = 64), 16 high-probability and 48 low-probability. Flanker congruency types (neutral, congruent, incongruent) were defined for the last element of all triplets, and in every occurrence of a given unique triplet, the last element had the same flanker congruency type. 37.5% of both high- and low-probability triplets (six and 18 unique triplets, respectively) were assigned to the

congruent type, another 37.5% were assigned to the incongruent type, while the remaining 25% (four and 12 unique triplets, respectively) became neutral.

During the assignment of flanker congruency types to high-probability triplets, we followed these criteria: minimum one, maximum two unique triplets of the same predictive association fell into the congruent and incongruent types, while one always fell into the neutral type (e.g., for the sequence of left – *r* – right – *r* – up – *r* – down – *r*, for triplets with left in the first position and right in the last position, left – up – right could have been congruent, left – right – right and left – down – right could have been incongruent, and left – left – right could have been neutral). In other words, a given predictive association between trial N and trial N-2 could have been congruent, incongruent, and neutral as well, preventing flanker congruency from being directly predictable based solely on the nonadjacent triplet structure.

During the assignment of flanker congruency types to low-probability triplets, we followed these criteria: minimum one, maximum two unique triplets of the same predictive association fell into the congruent and incongruent types, while one always fell into the neutral type (e.g., for the sequence of left – *r* – right – *r* – up – *r* – down – *r*, for triplets with left in the first position and up in the last position, left – up – up could have been congruent, left – right – up and left – down – up could have been incongruent, and left – left – up could have been neutral). Another criterion was to have trills and full repetitions within all flanker congruency types.

Afterwards, the above-described assignment steps were repeated twice more, resulting in three sequences that were at least partially different from one another. For all sequences, flanker congruency types were then partially mirrored: what was congruent became incongruent and vice versa, while neutral trials remained the same. Altogether, the flanker congruency structure was mapped onto the six unique ASRT base sequences in six distinctive ways (3 × 2; henceforth referred to as flanker-ASRT sequences). It is important to note once more that, although flanker congruency was defined using the triplet structure of the ASRT base sequences, it could not be predicted from the nonadjacent predictive association between the first and third triplet elements alone.

Selection – Following the assignment process, we statistically inspected the generated flanker-ASRT sequences and selected the most unbiased ones. During the selection process, we excluded those flanker-ASRT sequences that were characterized by 1) longer than three consecutive repetitions of the same flanker congruency type and/or 2) the unequal distribution of a flanker congruency type across response types (i.e., the four possible spatial directions of

the target stimulus) or longer-range time bins (i.e., within blocks and sets of five blocks). Second, the mirrored variants of these sequences were also excluded. Finally, we randomly selected and excluded additional flanker-ASRT sequence variations so that all six ASRT base sequences were represented in an equal number.

The final flanker-ASRT sequence pool contained six sequences and their mirrored variants (12 unique flanker-ASRT sequences altogether). Subsequently, we re-examined the sequence- and group-level characteristics of the remaining sequence files. Trills and repetitions within the low-probability triplet type (see above) were not examined here, as these were excluded from the main analysis. Due to the necessarily unequal number of trials within the high-probability vs. low-probability triplet types (62.5% vs. 37.5%) and within the incongruent/congruent vs. neutral flanker congruency types (37.5/37.5% vs. 25%), we conducted separate analyses along these dimensions. We used log-linear regressions to examine the distribution of flanker congruency types, with counts of a given flanker congruency type as a categorical dependent variable. In the case of congruent and incongruent trials, the regression model contained Sequence [1-12], Flanker [Congruent vs. Incongruent], and Response type [1-4] as independent variables. In other words, we aimed to determine whether the distribution of congruent vs. incongruent flanker congruency types differed across the four possible response types (target stimulus directions) and the 12 selected flanker-ASRT sequences. In the case of neutral trials, the model contained only Sequence [1-12] and Response type [1-4]: we tested whether the distribution of neutral trials differed across response types and flanker-ASRT sequences. To summarize, the goal of these analyses was to identify any significant distortion of the flanker congruency type assignment that might still be present in the selected flanker-ASRT sequences.

The results showed that in the case of high-probability trials, the assignment of congruent and incongruent flanker congruency types to the four possible response types varied across flanker-ASRT sequences (Tables S1, three-way interaction). To explore this interaction in depth, we conducted post-hoc Chi-squared tests, which revealed that the distortion was present in every sequence (all χ^2s ≥ 93.02, all *p*s $\leq .001$). Nevertheless, the distribution of congruent versus incongruent flanker congruency types did not differ across response types in the whole sample of flanker-ASRT sequences (nonsignificant Response type × Flanker interaction). Moreover, all sequences were used an equal number of times and in a counterbalanced order. Therefore, we did not treat this effect as a confounding factor. For low-probability trials, the distribution of congruent vs. incongruent trials was undistorted across response types and flanker-ASRT sequences (Table S2).

In the case of neutral trials, the distribution of high-probability trials across response types and flanker-ASRT sequences was undistorted (Table S3). For neutral low-probability trials, however, a significant main effect of Response type emerged (Table S4). To further explore it, we conducted a binomial test with a test value of 1/4 = 0.25. The post-hoc analysis revealed that in the whole sample of flanker-ASRT sequences, low-probability neutral trials appeared more frequently in position 2 (up; 27.8%, $p = .004$) and less frequently in position 4 (right; 22.5%, $p = .011$, all other ps $\geq .404$) than expected based on a uniform distribution. Nevertheless, despite the statistical significance of these differences, they are negligible, as they remained within a 5% deviation from the uniform distribution.

In summary, these analyses revealed some assignment-related imbalances in the high-probability congruent and incongruent trials, though these were not present when all flanker-ASRT sequences were considered together. The distribution of the remaining trial types appeared to be largely undistorted. In the experiment, flanker-ASRT sequences were assigned to participants pseudorandomly and counterbalanced across participants, reducing the likelihood that the obtained findings were driven by distributional tendencies specific to a particular sequence.

Table S1. Main effects and interactions observed in the log-linear regression for congruent vs. incongruent high-probability trials.

	df	Deviance	*p*
NULL			
Sequence	11	0.805	1.000
**Response type**	**3**	**12.144**	**.007**
Flanker	1	0.000	1.000
Sequence × Response type	33	14.620	.998
Sequence × Flanker	11	8.213	.694
Response type × Flanker	3	0.000	1.000
**Sequence × Response type × Flanker**	**33**	**1429.736**	**<.001**

Note. Significant ($p < .050$) effects are **bold-faced**.

Table S2. Main effects and interactions observed in the log-linear regression for congruent vs. incongruent low-probability trials.

	df	Deviance	*p*
NULL			
Sequence	11	2.736	.994
Response type	3	3.984	.263
Flanker	1	0.000	1.000
Sequence × Response type	33	19.550	.969
Sequence × Flanker	11	5.563	.901
Response type × Flanker	3	1.279×10^{-13}	1.000
Sequence × Response type × Flanker	33	18.782	.978

Table S3. Main effects and interactions observed in the log-linear regression for neutral high-probability trials.

	df	Deviance	*p*
NULL			
Sequence	11	9.290	.595
Response type	3	5.098	.165
Sequence × Response type	33	30.670	.584

Table S4. Main effects and interactions observed in the log-linear regression for neutral low-probability trials.

	df	Deviance	*p*
NULL			
Sequence	11	10.766	.463
**Response type**	**3**	**11.762**	**.008**
Sequence × Response type	33	11.421	1.000

Note. Significant ($p < .050$) effects are **bold-faced**.

2. Supplementary Results

Table S5. Estimated marginal means: Accuracy (error rate).

Trial type	Error rate (%)	*SE* (logit)
High-probability neutral	5.4	0.044
Low-probability neutral	6.8	0.054
High-probability congruent	3.6	0.043
Low-probability congruent	4.9	0.052
High-probability incongruent	8.3	0.030
Low-probability incongruent	11.1	0.036

2.1 Analysis of accuracy (error rate)

To analyze accuracy, calculated as error rate, a generalized linear mixed-effects model with binomial distribution (logit link) was fitted to responses coded as correct (0) or error (1) using the *glmer* function from the *lme4* package (Bates, Mächler, Bolker, & Walker, 2015) in R (R Core Team, 2020). The fixed effects included *Triplet* (high vs. low), *Flanker* (neutral, congruent, incongruent), and their two-way interaction. Both predictors were treated as categorical variables. Random intercepts were included for each participant × block combination. Model parameters were estimated using maximum likelihood with the Laplace approximation. The *bobyqa* optimizer was used with 100,000 iterations to improve convergence. *p*-values for fixed effects were derived from Wald *z*-tests as provided by *lme4*. Sum coding was applied, with *low* (Triplet) and *incongruent* (Flanker) set as reference levels. Pairwise comparisons were conducted using the *emmeans* package (Lenth, 2022) with Tukey adjustment for *p*-values. Figures were generated with the *ggplot2* package (Wickham, 2016). The schematic structure of the model is summarized as: errors ~ Triplet × Flanker + (1 | participant:block).

The logistic mixed-effects model (see Table S6) revealed a significant statistical learning effect (main effect of Triplet), with fewer errors on predictable (high-probability) than on unpredictable (low-probability) trials (5.5% vs. 7.0%; $\beta = -0.15$, $SE = 0.02$, $z = -8.66$, $p < .001$). A strong flanker congruency effect also emerged (main effect of Flanker), with congruent trials yielding significantly fewer errors than incongruent ones (4.3% vs. 9.7%; $\beta = -0.42$, $SE = 0.03$, $z = -16.37$, $p < .001$). The neutral–incongruent contrast was not significant as represented by the corresponding contrast-coded parameter in the mixed-effects model ($\beta = -0.04$, $SE = 0.03$, $z = -1.59$, $p = .112$). In contrast, a direct pairwise comparison of the estimated marginal means, averaged across triplet probability, revealed a robust neutral–incongruent

difference (6.0% vs. 9.7%; $z = -12.40$, $p < .001$), indicating fewer errors on neutral than incongruent trials. This discrepancy reflects the different quantities tested by the contrast-coded model parameter and the direct marginal pairwise comparison. In addition, neutral trials elicited significantly more errors than congruent trials (6.0% vs. 4.3%; $z = 8.06$, $p < .001$).

Crucially, unlike the RTs, the Triplet × Flanker interaction was not significant for error rates. Specifically, the statistical learning effect did not significantly differ across the congruency conditions. The corresponding contrast-coded model parameters were nonsignificant for neutral vs. incongruent ($\beta = 0.03$, $SE = 0.03$, $z = 0.98$, $p = .328$) and congruent vs. incongruent trials ($\beta = -0.01$, $SE = 0.03$, $z = -0.50$, $p = .615$). Direct comparisons of the statistical learning effects based on estimated marginal means were also nonsignificant (neutral vs. incongruent: $z = -0.94$, $p = .615$; congruent vs. incongruent: $z = 0.01$, $p = 1.000$; neutral vs. congruent: $z = -0.82$, $p = .692$). Likewise, the flanker congruency effect (incongruent > congruent) did not differ significantly between predictable and unpredictable trials ($z = 0.01$, $p = 1.000$). Descriptively, the flanker congruency effect on the response scale was 4.7% for predictable trials and 6.2% for unpredictable trials (difference = 1.5%; see also Table S5). Although the statistical learning effect was somewhat larger in the incongruent condition than in neutral or congruent ones, these differences were not statistically significant (1.4%, 1.3%, and 2.8% error reduction for the neutral, congruent, and incongruent trials, respectively; see also Table S5). Importantly, however, statistical learning effects remained significantly above zero in all flanker congruency conditions (all ps $\leq .0002$), confirming the robustness of the effect. These results are presented in Figure S1.

Table S6. Summary of the logistic mixed-effects model testing the effects of predictability and flanker congruency on errors.

Terms	β	*SE*	*95% CI*	*z*	*p*
(Intercept)	-2.703	0.020	-2.743 – -2.663	-133.165	**<.001**
Triplet (High vs. Low)	-0.151	0.017	-0.185 – -0.117	-8.657	**<.001**
Neutral vs. Incongruent	-0.042	0.026	-0.093 – 0.010	-1.587	.112
Congruent vs. Incongruent	-0.422	0.026	-0.472 – -0.371	-16.367	**<.001**
Triplet × Neutral vs. Incongruent	0.026	0.026	-0.026 – 0.077	0.978	.328
Triplet × Congruent vs. Incongruent	-0.013	0.026	-0.064 – 0.038	-0.503	.615
**Random Effects**					
σ^2	3.290				
$\tau_{00\ ID:block}$	0.074				
ICC	0.022				
N_{ID}	36				
N_{block}	30				
Observations	64000				
Marginal R^2 / Conditional R^2	0.048 / 0.069				

Note. Significant (p < .050) effects are **bold-faced**. Coding scheme: sum coding. Reference levels: low (Triplet) and incongruent (Flanker). β values represent log-odds coefficients. *SE*: standard error; *CI*: confidence interval.

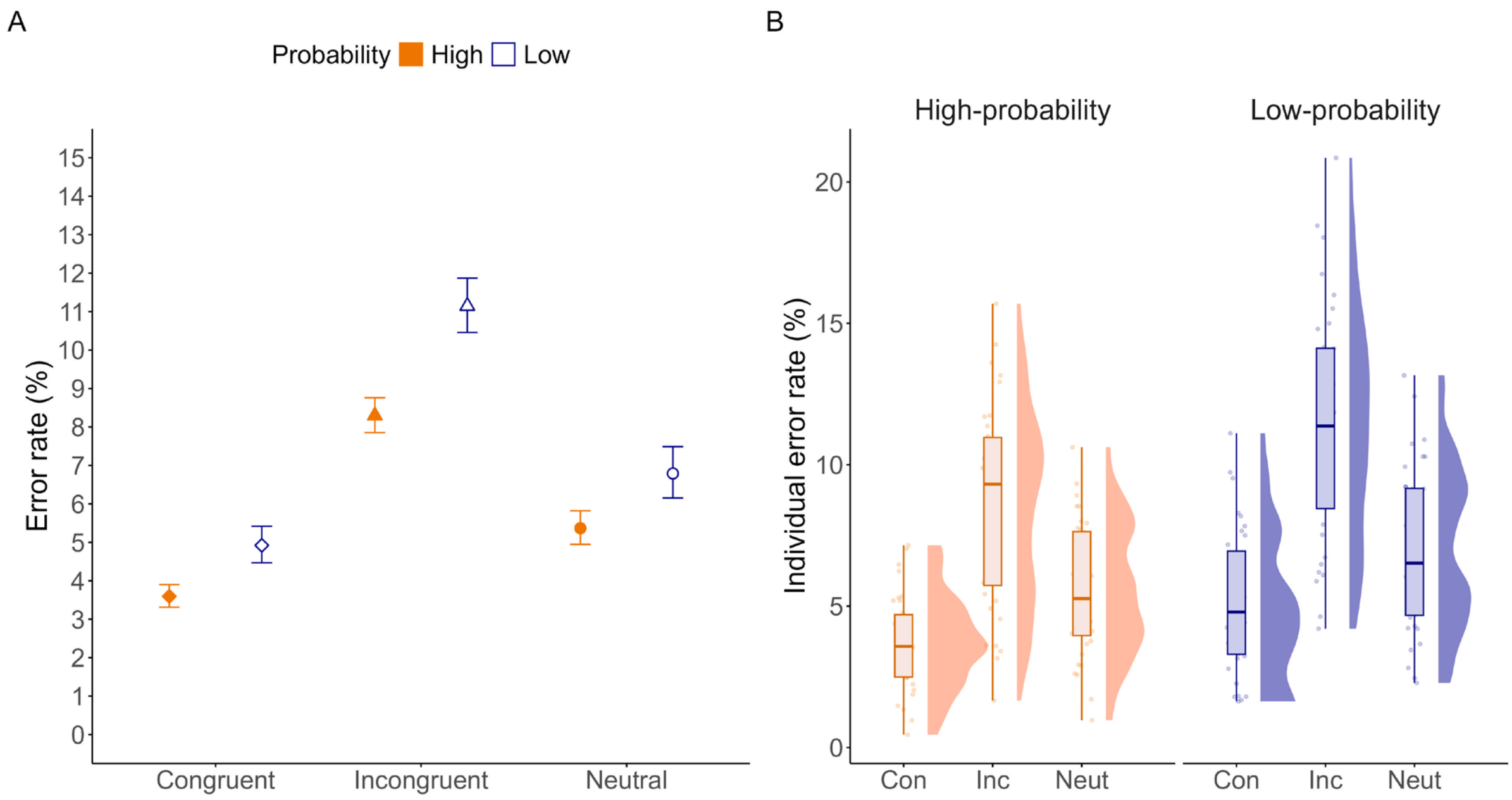

Figure S1. Main task performance across probability and flanker congruency conditions. (A) Mean error rate (%) across probability conditions, with orange representing high-probability (predictable) trials and blue representing low-probability (unpredictable) trials, shown separately for each flanker congruency type on the x-axis (congruent, incongruent, neutral). Error rates were lower for high-probability than for low-probability trials, reflecting statistical learning. Error rates were higher on incongruent than on congruent or neutral trials, reflecting the flanker congruency effect. Unlike RTs, no significant interaction emerged between probability and flanker congruency. Error bars denote 95% confidence intervals. **(B)** Individual condition-level error rates (jittered, semi-transparent points), overlaid with half-eye density plots (colored areas) that illustrate the full distribution of the data across probability and flanker congruency types (Con = congruent, Inc = incongruent, Neut = neutral). Central boxplots indicate the median (horizontal line) and interquartile range (box edges), with whiskers extending to 1.5 times the interquartile range.

2.2 Analysis of RTs controlling for repetition priming

A linear mixed-effects model was fitted to RTs of correctly responded trials using the *lmer* function, as in the main analysis. The fixed effects included *Triplet* (high vs. low), *Flanker* (neutral, congruent, incongruent), *Distractor Repetition* (changed distractors vs. repeated distractors), *Response Repetition* (changed response vs. repeated response), and all two-way, three-way, and four-way interactions. All predictors were treated as categorical variables. Random intercepts were included for each participant × block combination. Sum coding was applied, with *low* (Triplet), *incongruent* (Flanker), *repeated distractor* (Distractor Repetition), and *repeated response* (Response Repetition) set as reference levels. Modeling and further analyses followed the same procedures as in the simpler RT model reported in the main text.

The schematic structure of the model was: RTs ~ Triplet × Flanker × Distractor Repetition × Response Repetition + (1 | participant:block).

The model (see Table S7) revealed significant statistical learning (main effect of Triplet, $\beta = -3.01$, $SE = 0.34$, $t = -8.90$, $p < .001$) and flanker congruency effects (neutral vs. incongruent: $\beta = -8.83$, $SE = 0.49$, $t = -17.95$, $p < .001$; congruent vs. incongruent: $\beta = -10.92$, $SE = 0.47$, $t = -23.25$, $p < .001$), replicating the simpler RT model. Predictable (high-probability) trials were faster than unpredictable (low-probability) ones, and both neutral and congruent trials were faster than incongruent ones.

Importantly, the Triplet × Flanker interaction remained significant ($\beta = 1.48$, $SE = 0.47$, $t = 3.14$, $p = .002$). This interaction was numerically stronger than in the simpler model and did not further interact with distractor or response repetition (nonsignificant three-way and four-way interactions involving Distractor Repetition and Response Repetition, all ps $\geq .163$). Specifically, the statistical learning effect was larger for incongruent than for congruent trials (8.75 msec vs. 3.05 msec, difference = 5.70 msec; $t = -3.50$, $p = .001$). Equivalently, the flanker congruency effect (incongruent > congruent) was reduced for predictable relative to unpredictable trials (27.81 msec vs. 33.51 msec, difference = 5.70 msec). The statistical learning effect did not differ significantly between incongruent and neutral trials ($p = .297$) or between congruent and neutral trials ($p = .136$), similarly to the simpler model. Again, statistical learning effects remained significantly above zero in all flanker congruency conditions (all ps $< .010$; 6.23 msec, 3.05 msec, and 8.75 msec for the neutral, congruent, and incongruent trials, respectively). Together, these results indicate that the interaction between statistical learning and flanker congruency is robust after accounting for short-term repetition priming.

Repetition effects themselves were relatively small but systematic. Distractor repetition significantly facilitated responses (main effect of Distractor Repetition, $\beta = 1.09$, $SE = 0.34$, $t = 3.21$, $p < .001$), whereas response repetition tended to slow them (main effect of Response Repetition, $\beta = -0.58$, $SE = 0.34$, $t = -1.72$, $p = .085$). Crucially, the significant Distractor Repetition × Response Repetition interaction ($\beta = -1.82$, $SE = 0.34$, $t = -5.37$, $p < .001$) revealed that response repetition slowed RTs when the distractors changed ($t = -6.82$, $p < .001$) but facilitated RTs when distractors repeated ($t = 2.15$, $p = .032$).

More importantly, the joint influence of distractor and response repetition varied with flanker congruency type (significant Flanker × Distractor Repetition × Response Repetition interaction; neutral vs. incongruent trials: $\beta = 2.04$, $SE = 0.49$, $t = 4.14$, $p < .001$; congruent vs. incongruent trials: $\beta = -1.89$, $SE = 0.47$, $t = -4.00$, $p < .001$; see Figure S2 and also Table S8). For congruent vs. incongruent trials, the flanker congruency effect (incongruent minus

congruent difference) was largest when the response repeated but the distractors changed (37.8 msec). This was significantly greater than in the full alteration condition (i.e., both distractor and response change, 32.7 msec; $t = 3.32$, $p = .005$) and in the distractor repetition-only condition (20.0 msec; $t = –7.77$, $p < .001$). The congruent vs. incongruent flanker congruency effect under distractor repetition only was also smaller than under full alteration ($t = –6.69$, $p < .001$) and full repetition (i.e., both distractor and response repetition, 32.7 msec; $t = 4.56$, $p < .001$).

A similar but slightly different pattern was observed for neutral vs. incongruent trials: The effect (incongruent minus neutral difference) was again largest when the response repeated but distractors changed (40.7 msec), exceeding all other conditions (full alteration, 28.0 msec; $t = 7.19$, $p < .001$; full repetition, 24.8 msec; $t = –5.58$, $p < .001$; and distractor repetition only, 20.8 msec; $t = –9.06$, $p < .001$). The effect under distractor repetition only was also smaller than under full alteration ($t = –3.95$, $p < .001$). Further contrasts for the neutral vs. congruent comparison are reported in Table S8, indicating that the facilitatory effect of congruent trials was present only in full repetition and full alteration conditions.

Comparing RTs by flanker type (Table S9) further illuminates these interactions. Incongruent RTs were slowest when only the response repeated and fastest when only the distractors repeated (404 msec vs. 388 msec; $t = –9.70$, $p < .001$). For congruent trials, RTs were fastest under full repetition (359 msec) and full alteration (361 msec), while both response-only and distractor-only repetition slowed them down (366 msec and 368 msec, all *p*s < .001). Neutral trials were comparatively less sensitive to repetition priming: Distractor-only repetition slowed responses relative to response-only repetition (367 msec vs. 363 msec; $t = 2.75$, $p = .030$). These differences together account for the observed modulations of the flanker congruency effects across repetition priming conditions.

Finally, repetition priming also influenced statistical learning. Distractor repetition tended to increase the statistical learning effect (Triplet × Distractor Repetition, $\beta = 0.61$, $SE = 0.34$, $t = 1.80$, $p = .072$), whereas response repetition significantly reduced it (Triplet × Response Repetition, $\beta = –0.86$, $SE = 0.34$, $t = –2.52$, $p = .012$). Specifically, response repetition slowed RTs to predictable (high-probability) trials ($t = –3.67$, $p < .001$) but had no effect on unpredictable (low-probability) trials ($t = 0.50$, $p = .617$), thereby decreasing the RT difference between them and weakening the statistical learning effect. All these results are presented in Figure S2.

Table S7. Summary of the linear mixed-effects model testing the effects of predictability, flanker congruency, and repetition priming on RTs.

Terms	β	*SE*	*95% CI*	*t*	df	*p*
(Intercept)	374.483	0.989	372.542 – 376.424	378.499	1219.552	**<.001**
Triplet (High vs. Low)	-3.005	0.338	-3.667 – -2.343	-8.900	58742.587	**<.001**
Neutral vs. Incongruent	-8.829	0.492	-9.793 – -7.864	-17.947	58833.164	**<.001**
Congruent vs. Incongruent	-10.916	0.469	-11.836 – -9.996	-23.251	58787.958	**<.001**
Distractor Repetition (Change vs. Repetition)	1.092	0.340	0.425 – 1.759	3.208	58858.645	**.001**
Response Repetition (Change vs. Repetition)	-0.580	0.337	-1.241 – 0.081	-1.720	58719.378	.085
Triplet × Neutral vs. Incongruent	-0.112	0.490	-1.072 – 0.848	-0.228	58772.640	.820
Triplet × Congruent vs. Incongruent	1.481	0.471	0.558 – 2.405	3.143	58844.665	**.002**
Triplet × Distractor Repetition	0.612	0.340	-0.054 – 1.277	1.801	58826.720	.072
Neutral vs. Incongruent × Distractor Repetition	-2.444	0.495	-3.415 – -1.473	-4.934	58930.016	**<.001**
Congruent vs. Incongruent × Distractor Repetition	-0.919	0.471	-1.842 – 0.005	-1.950	58837.408	.051
Triplet × Response Repetition	-0.856	0.340	-1.523 – -0.190	-2.517	58846.265	**.012**
Neutral vs. Incongruent × Response Repetition	1.254	0.490	0.292 – 2.215	2.556	58790.339	**.011**
Congruent vs. Incongruent × Response Repetition	1.678	0.475	0.746 – 2.609	3.529	58962.221	**<.001**
Distractor Repetition × Response Repetition	-1.821	0.339	-2.485 – -1.157	-5.373	58798.629	**<.001**
Triplet × Neutral vs. Incongruent × Distractor Repetition	-0.084	0.491	-1.047 – 0.879	-0.171	58810.086	.864
Triplet × Congruent vs. Incongruent × Distractor Repetition	-0.655	0.470	-1.577 – 0.266	-1.394	58808.042	.163
Triplet × Neutral vs. Incongruent × Response Repetition	0.065	0.497	-0.908 – 1.039	0.132	58964.482	.895
Triplet × Congruent vs. Incongruent × Response Repetition	-0.641	0.476	-1.575 – 0.293	-1.346	58988.778	.178
Triplet × Distractor Repetition × Response Repetition	0.488	0.342	-0.182 – 1.157	1.427	58910.910	.153
Neutral vs. Incongruent × Distractor Repetition × Response Repetition	2.036	0.492	1.072 – 2.999	4.140	58827.649	**<.001**

Congruent vs. Incongruent × Distractor Repetition × Response Repetition	-1.889	0.473	-2.816 – -0.963	-3.997	58886.332	**<.001**
Triplet × Neutral vs. Incongruent × Distractor Repetition × Response Repetition	0.061	0.490	-0.899 – 1.020	0.124	58766.229	.901
Triplet × Congruent vs. Incongruent × Distractor Repetition × Response Repetition	0.580	0.471	-0.343 – 1.503	1.231	58831.829	.218
**Random Effects**						
σ^2	3278.767					
$\tau_{00\ ID:block}$	934.161					
ICC	0.222					
N_{ID}	36					
N_{block}	30					
Observations	59753					
Marginal R^2 / Conditional R^2	0.050 / 0.261					

Note. Significant ($p < .050$) effects are **bold-faced**. Coding scheme: sum coding. Reference levels: low (Triplet), incongruent (Flanker), repeated distractor (Distractor Repetition), and repeated response (Response Repetition). *SE*: standard error; *CI*: confidence interval. In the case of Distractor/Response Repetition, positive estimates indicate that the presence of repetition facilitates responses by reducing RTs (as opposed to distractor/response change), whereas negative estimates indicate that the presence of repetition produces interference by increasing RTs (as opposed to distractor/response change).

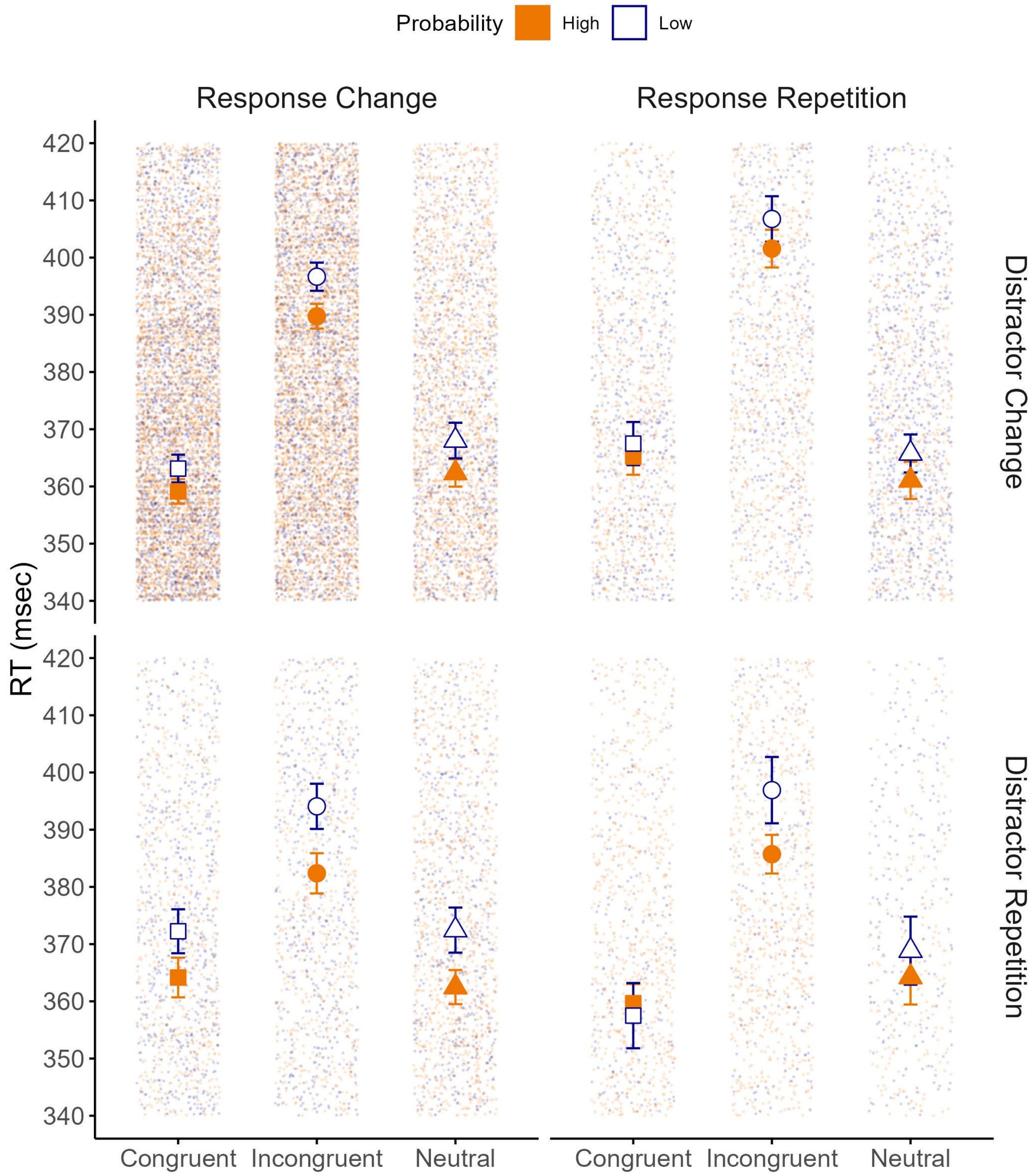

Figure S2. RTs by flanker congruency, triplet type, and repetition priming. Mean RTs as a function of flanker congruency (congruent, incongruent, neutral), triplet type (high- vs. low-probability), distractor repetition (repetition vs. change), and response repetition (repetition vs. change). Data points represent condition-wise group means estimated from the linear mixed-effects model, with error bars denoting 95% confidence intervals. Background dots show individual trial-level data (jittered for visibility). Facets separate the four repetition priming conditions (distractor repetition vs. change × response repetition vs. change). Full alteration is the combination of distractor and response change (top-left facet); full repetition is the combination of distractor and response repetition (bottom-right facet). The figure illustrates that the statistical learning effect (faster RTs for high- vs. low-probability triplets) and the flanker congruency effects (slower responses for incongruent vs. congruent and neutral trials) were both robust, and that their interaction remained significant across repetition priming conditions.

The interaction could be described either as a larger statistical learning effect in incongruent relative to congruent trials or as a reduced flanker congruency effect for predictable relative to unpredictable trials. Repetition priming modulated flanker congruency effects and the statistical learning effect separately.

Table S8. Follow-up of the Flanker × Distractor Repetition × Response Repetition interaction: Pairwise comparisons of the flanker congruency effects (Incongruent vs. Congruent, Incongruent vs. Neutral) across the factor level combinations of repetition priming (Distractor Repetition × Response Repetition).

Contrast			Estimate	*SE*	df	*t*	*p*
INCONGRUENT VS. CONGRUENT DIFFERENCE							
Response repetition	–	Full alteration	5.731	1.73	58849	3.321	**.005**
Distractor repetition	–	Full alteration	-12.049	1.80	58905	-6.692	**<.001**
Distractor repetition	–	Response repetition	-17.780	2.29	58959	-7.77	**<.001**
Full repetition	–	Full alteration	0.655	2.33	58872	0.281	.992
Full repetition	–	Response repetition	-5.076	2.71	58828	-1.874	.239
Full repetition	–	Distractor repetition	12.704	2.79	59005	4.557	**<.001**
INCONGRUENT VS. NEUTRAL DIFFERENCE							
Response repetition	–	Full alteration	12.734	1.77	58872	7.194	**<.001**
Distractor repetition	–	Full alteration	-7.250	1.84	58829	-3.945	**<.001**
Distractor repetition	–	Response repetition	-19.984	2.20	58908	-9.064	**<.001**
Full repetition	–	Full alteration	-3.244	2.59	58851	-1.251	.594
Full repetition	–	Response repetition	-15.978	2.86	58888	-5.579	**<.001**
Full repetition	–	Distractor repetition	4.006	2.90	58837	1.382	.511
NEUTRAL VS. CONGRUENT DIFFERENCE							
Response repetition	–	Full alteration	-7.003	1.72	58875	-4.067	**<.001**
Distractor repetition	–	Full alteration	-4.799	1.81	58835	-2.647	**.041**
Distractor repetition	–	Response repetition	2.204	2.15	58912	1.024	.735
Full repetition	–	Full alteration	3.899	2.57	58850	1.516	.428

Full repetition	–	Response repetition	10.901	2.82	58894	3.864	**<.001**
Full repetition	–	Distractor repetition	8.698	2.87	58838	3.031	**.013**

Note. Significant ($p < .050$) differences are **bold-faced**. Minus sign indicates that the flanker congruency effects (and the neutral vs. congruent contrast) are compared between each level combination of the repetition priming factors (Distractor Repetition × Response Repetition). The meanings of the factor level combinations are as follows. Response repetition: Response repetition and Distractor change. Full alteration: Distractor change and Response change. Distractor repetition: Distractor repetition and Response change. Full repetition: Distractor repetition and Response repetition. Pairwise comparisons were conducted using the *emmeans* package with the Tukey method for *p*-value adjustments.

Table S9. Follow-up of the Flanker × Distractor Repetition × Response Repetition interaction: Pairwise comparisons of each flanker type (Neutral, Congruent, and Incongruent) across the factor level combinations of repetition priming (Distractor Repetition × Response Repetition).

Contrast			Estimate	*SE*	df	*t*	*p*
NEUTRAL							
Full alteration	–	Distractor repetition	-2.275	1.32	58897	-1.722	.312
Full alteration	–	Response repetition	1.777	1.24	58837	1.429	.481
Full alteration	–	Full repetition	-1.356	1.99	58818	-0.683	.904
Distractor repetition	–	Response repetition	4.052	1.47	58967	2.754	**.030**
Distractor repetition	–	Full repetition	0.918	2.12	58749	0.434	.973
Response repetition	–	Full repetition	-3.134	2.09	58869	-1.5	.438
CONGRUENT							
Full alteration	–	Distractor repetition	-7.074	1.25	58855	-5.658	**<.001**
Full alteration	–	Response repetition	-5.225	1.18	58826	-4.417	**<.001**
Full alteration	–	Full repetition	2.542	1.63	58835	1.562	.401
Distractor repetition	–	Response repetition	1.848	1.57	58862	1.177	.641
Distractor repetition	–	Full repetition	9.616	1.93	58911	4.97	**<.001**
Response repetition	–	Full repetition	7.768	1.88	58841	4.123	**<.001**
INCONGRUENT							
Full alteration	–	Distractor repetition	4.975	1.29	58848	3.869	**<.001**
Full alteration	–	Response repetition	-10.957	1.25	58823	-8.749	**<.001**

Full alteration	–	Full repetition	1.887	1.66	58835	1.137	.667
Distractor repetition	–	Response repetition	-15.932	1.64	58861	-9.702	**<.001**
Distractor repetition	–	Full repetition	-3.088	1.98	58906	-1.561	.401
Response repetition	–	Full repetition	12.844	1.95	58835	6.594	**<.001**

Note. Significant ($p < .050$) differences are **bold-faced**. Minus sign indicates that RTs for each flanker type are compared between each level combination of the repetition priming factors (Distractor Repetition × Response Repetition). The meanings of the factor level combinations are as follows. Full alteration: Distractor change and Response change. Distractor repetition: Distractor repetition and Response change. Response repetition: Response repetition and Distractor change. Full repetition: Distractor repetition and Response repetition. Pairwise comparisons were conducted using the *emmeans* package with the Tukey method for *p*-value adjustments.

Supplementary References

Bates, D., Mächler, M., Bolker, B., & Walker, S. (2015). Fitting Linear Mixed-Effects Models Using lme4. *Journal of Statistical Software, 67*(1), 1-48. doi:10.18637/jss.v067.i01

Lenth, R. V. (2022). emmeans: Estimated Marginal Means, aka Least-Squares Means. R package version 1.7.5. Retrieved from https://CRAN.R-project.org/package=emmeans

R Core Team (2020). R: A language and environment for statistical computing. Vienna, Austria: R Foundation for Statistical Computing. Retrieved from http://www.R-project.org/

Wickham, H. (2016). *ggplot2: Elegant Graphics for Data Analysis*: Springer-Verlag New York.
